# PACAP centrally mediates emotional stress-induced corticosterone responses in mice

**DOI:** 10.3109/10253890.2010.544345

**Published:** 2011-03-27

**Authors:** Naohiro Tsukiyama, Yoko Saida, Michiya Kakuda, Norihito Shintani, Atsuko Hayata, Yoshiko Morita, Mamoru Tanida, Minako Tajiri, Keisuke Hazama, Katsuya Ogata, Hitoshi Hashimoto, Akemichi Baba

**Affiliations:** 1 Laboratory of Molecular Neuropharmacology, Graduate School of Pharmaceutical Sciences, Osaka University, 1-6 Yamadaoka, Suita, Osaka 565-0871, Japan; 2 Center for Child Mental Development, United Graduate School of Child Development, Osaka University, Kanazawa University and Hamamatsu University School of Medicine, 2-2 Yamadaoka, Suita, Osaka 565-0871, Japan; 3 Department of Biomedical Sciences, College of Life Sciences, Ritsumeikan University, 1-1-1 Nojihigashi, Kusatsu, Shiga 525-8577, Japan; 4 Department of Molecular Pharmaceutical Science, Graduate School of Medicine, 2-2 Yamadaoka, Suita, Osaka 565-0871, Japan; 5 Hyogo University of Health Sciences, 1-3-6 Minatojima, Chuo-ku, Kobe, Hyogo 650-8530, Japan

**Keywords:** Corticosterone, hypothalamic paraventricular nucleus, hypothalamic–pituitary–adrenal axis, medial amygdala, PACAP, stress

## Abstract

Pituitary adenylate cyclase-activating polypeptide (PACAP) is a pleiotropic neuropeptide widely distributed in the nervous system. Recently, PACAP was shown to be involved in restraint stress-induced corticosterone release and concomitant expression of the genes involved in hypothalamic–pituitary–adrenal (HPA) axis activation. Therefore, in this study, we have addressed the types of stressors and the levels of the HPA axis in which PACAP signaling is involved using mice lacking PACAP (PACAP^−/−^). Among four different types of stressors, open-field exposure, cold exposure, ether inhalation, and restraint, the corticosterone response to open-field exposure and restraint, which are categorized as emotional stressors, but not the other two, was markedly attenuated in PACAP^−/−^ mice. Peripheral administration of corticotropin releasing factor (CRF) or adrenocorticotropic hormone induced corticosterone increase similarly in PACAP and wild-type mice. In addition, the restraint stress-induced c-Fos expression was significantly decreased in the paraventricular nucleus (PVN) and medial amygdala (MeA), but not the medial prefrontal cortex, in PACAP^−/−^ mice. In the PVN of PACAP^−/−^ mice, the stress-induced c-Fos expression was blunted in the CRF neurons. These results suggest that PACAP is critically involved in activation of the MeA and PVN CRF neurons to centrally regulate the HPA axis response to emotional stressors.

## Introduction

Pituitary adenylate cyclase-activating polypeptide (PACAP) is a neuropeptide that belongs to the vasoactive intestinal polypeptide/secretin/glucagon family and has an ability to stimulate adenylate cyclase in anterior pituitary cells and to increase the release of pituitary hormone including corticotropin (ACTH) (Miyata et al. 1989; Vaudry et al. 2009). Both PACAP and the PACAP receptor subtype PAC1 are highly expressed in the hypothalamus, the pituitary gland, and the adrenal gland, and promote the production and release of hormones in these tissues (Vaudry et al. 2009). It was reported that centrally administered PACAP induces stress-related behaviors and systemic injection of PACAP increases plasma corticosterone and epinephrine levels (Agarwal et al. 2005). In contrast, it was shown that chronic stress increases PACAP and brain-derived neurotrophic factor mRNA expression in the bed nucleus of the stria terminalis in the extended amygdala (Hammack et al. 2009).

Previously, we developed mice that lack the PACAP gene (PACAP^−/−^) and showed that these mice exhibited remarkable behavioral changes, including hyperactivity, novelty-seeking behavior, sensory-motor deficits, and depression-like behavior (Hashimoto et al. 2001, 2009; Tanaka et al. 2006), suggesting a role for altered PACAP-mediated signaling pathways in certain psychiatric conditions. In addition, PACAP^−/−^ mice showed an impaired sympathoadrenal response to insulin-induced metabolic stress (Hamelink et al. 2002) as well as a decreased corticosterone response to the organometal neurotoxin trymethyltin (Morita et al. 2006) and bright light exposure at night (Hatanaka et al. 2008). Recently, Stroth and Eiden (2010) demonstrated that PACAP is involved in restraint stress-induced corticosterone release and concomitant expression of the genes involved in hypothalamic–pituitary–adrenal (HPA) axis activation in their PACAP-deficient mice line. Moreover, we reported an association between single nucleotide polymorphisms in the genes for PACAP and its receptor PAC1 and schizophrenia (Hashimoto et al. 2007) as well as between the PACAP gene and depression (Hashimoto et al. 2010). As it is widely recognized that stress acts as a predisposing factor in psychiatric disorders, PACAP may play an important role in the perception of stress and adaptation to it, of which failure may become one of the causes of psychiatric disorders.

Thus, accumulating evidence supports the idea that PACAP is involved in stress-related response. In this study, we have therefore addressed the types of stressors and the levels of the HPA axis in which PACAP signaling is involved using PACAP^−/−^ mice.

## Materials and methods

### Animals

Generation of PACAP^−/−^ mice by a gene targeting technique has been reported previously (Hashimoto et al. 2001). The null mutation was backcrossed onto the genetic background of Crlj:CD1 mice (Charles River, Tokyo, Japan) at least 10 times. All wild-type control and PACAP^−/−^ mice used were obtained from the intercross of animals heterozygous for the mutant PACAP gene, and experiments were conducted with naïve male mice of 10–17 weeks of age. Mice were housed in a temperature (22 ± 1°C) and light-controlled room with a 12 h light/12h dark cycle (lights on from 08:00 to 20:00), and allowed free access to water and food (CE-2; Crea Japan Inc., Tokyo, Japan), except during behavioral testing. All animal experiments were carried out in accordance with protocols approved by the Animal Research Committee of Osaka University, Japan.

### Stress treatments

Mice were individually housed at least for 1 week before experiments. For restraint stress, mice were individually restrained for 30 min to 6 h in ventilated 50 ml polypropylene tubes (Falcon, Becton Dickinson, Lincoln, NJ, USA). For novel-environment-exposure stress, mice were placed in a circular open-field apparatus (60 cm in diameter, 30 cm deep, illuminated with white light of 100 lux) and left for 1 h. In a pilot study, we observed that PACAP^−/−^ mice showed greater hypothermia after a 4-h cold exposure compared with wild-type mice, when they were food deprived (data not shown). For cold-exposure stress, therefore, mice cages with water were carefully transferred into a cold chamber at 4°C or a chamber at 22°C following 2-h food deprivation, and left for 4 h. For ether-inhalation stress, mice were individually placed for 90 s in a 1 L-glass jar where a full-dose of ethyl ether was poured. Thereafter, mice were brought back to their home cages, and the duration of loss of righting reflex was measured.

### Corticosterone measurements

Mice were sacrificed at each time-point indicated, and trunk blood was collected into 1.5 ml plastic tubes containing 5 µl of 0.5 M EDTA. Samples were immediately centrifuged and heated at 60°C for 30 min, and the plasma was stored at −80°C until assayed. Corticosterone levels were determined with the Rat Corticosterone ^125^I Biotrack Assay System (GE Healthcare Bio-Sciences Corp., Piscataway, NJ, USA). In the corticotropin releasing factor (CRF) or ACTH challenge test, mice were administrated intraperitoneally with either vehicle (phosphate-buffered saline containing 0.3% (w/v) bovine serum albumin), 0.05–0.1 µg/mouse of CRF (Bachem, Torrence, CA, USA), or 1–10µg/kg body weight of ACTH (Bachem). At 30min after injection, trunk blood was collected and plasma corticosterone levels were then determined. In the case of ACTH, dexamethasone (Sigma-Aldrich Co., St Louis, MO, USA) was subcutaneously injected at 0.1 mg/kg body weight 30 min before ACTH injection to suppress endogenous ACTH secretion.

### Immunohistochemistry and quantitative analysis of immunostained sections

After restraint stress for 2 h, mice were sacrificed, while control mice were sacrificed without restraint stress, and c-Fos expression was analyzed immunohistochemically as described previously (Hatanaka et al. 2008). Briefly, 20 µm thick frontal sections containing the medial prefrontal cortex (mPFC), hypothalamic para-ventricular nucleus (PVN), and medial amygdala (MeA) were cut and processed for immunofluorescent staining. For double immunofluorescent staining, sections were incubated with anti-c-Fos mouse monoclonal antibody (1:1000 dilution; Santa Cruz, CA, USA) and anti-CRF rabbit polyclonal antibody (1:1000 dilution; Bachem) or anti-arginine vasopressin (AVP) rabbit polyclonal antibody (1:5000 dilution; Bachem) as primary antibodies, and then with species-specific fluorophore-conjugated secondary antibodies (c-Fos, Texas Red anti-mouse IgG, Adcam, Cambridge, UK, 1:500 dilution; CRF and AVP, Alexa 488-conjugated anti-rabbit IgG, 1:500 dilution, Invitrogen, Carlsbad, CA, USA).

Double-immunofluorescent-stained slices were photographed with a fluorescence microscope (Biozero BZ-9000, Keyence, Osaka, Japan), and the numbers of cells positive for c-Fos, CRF, and AVP were counted using Photoshop software (Adobe, San Jose, CA, USA). In addition, c-Fos/CRF or c-Fos/AVP double positive cells were counted manually in merged images. For each brain, the numbers of cells were averaged over 4–6 brain sections to determine the average number in each area. These quantitative analyses were performed by experienced observers blinded to the mouse genotype and treatment, and the anatomical localization was aided by the use of adjacent Nissl-stained sections and the illustrations in a stereotaxic atlas (Franklin and Paxinos 1997).

### Statistical analysis

Statistical analysis was performed using Statview software (SAS Institute, Cary, NC, USA). Statistically significant differences were assessed by ANOVA, followed by *post hoc* Mann–Whitney *U*-test or Tukey multiple comparison test, where applicable. All values are presented as the mean ± SEM. Statistical significance was assigned at *P* < 0.05.

## Results

### Corticosterone response to various types of stressors in PACAP2^−/−^ mice

**Figure 1:**
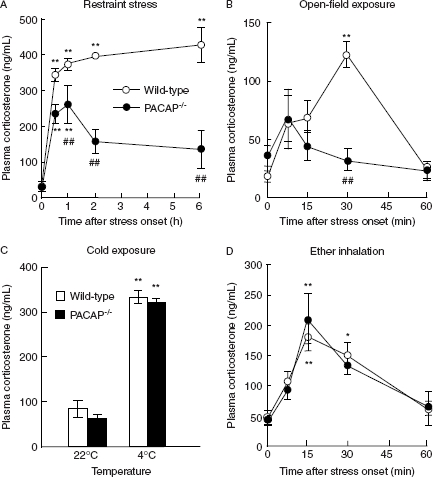
Impairment of corticosterone response to restraint stress and open-field exposure in PACAP^−/−^ mice. After restraint (A), open-field exposure (B), cold exposure (C), or ether-inhalation (D) stress, plasma corticosterone levels were determined in PACAP^−/−^ (closed symbols) and wild-type (open symbols) mice (*n* = 3–8 per group). **P < 0.01 compared with time 0, except for (C), 22°C, in the same genotype; ##*P* < 0.01 compared with wild-type mice.

We firstly examined the corticosterone responses in PACAP^−/−^ mice following four different types of acute stressors, restraint stress, open-field exposure, cold exposure, and ether inhalation (Figure 1). In accord with previous report (Stroth and Eiden 2010), PACAP^−/−^ mice exhibited a significant impairment in plasma corticosterone response to restraint stress (Figure 1A). Open-field exposure did not significantly increase corticosterone levels in PACAP^−/−^ mice, although wild-type mice showed a remarkable increase in corticosterone by approximately fivefold at 30 min after exposure (Figure 1B). In sharp contrast, cold exposure (Figure 1C) and ether inhalation (Figure 1D) increased plasma corticosterone levels similarly in PACAP^−/−^ and wild-type mice. In addition, the time of ether-induced loss of righting reflex, an indicator of ether effect on the central nervous system, did not differ between PACAP^−/−^ and wild-type mice (wild-type: 67.3 ± 4.7 s, *n* = 28; PACAP^−/−^ : 76.5 ± 5.7 s, *n* = 24, not significant).

### CRF- and ACTH-induced corticosterone release in PACAP^−/−^ mice

**Figure 2:**
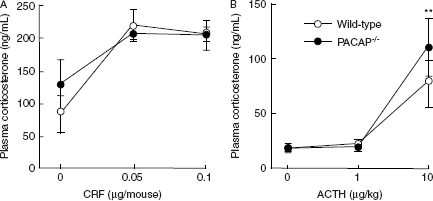
CRF- and ACTH-induced increase in plasma corticosterone levels in PACAP^−/−^ mice. Thirty minutes after injection of CRF (A) or ACTH (B), the plasma corticosterone levels were determined in PACAP^−/−^ (closed circles) and wild-type (open circles) mice (*n* = 3–6 per group). **P < 0.01 compared with 0 µg/kg.

In order to investigate whether the function of the pituitary or adrenal cortex are altered in terms of corticosterone release in PACAP^−/−^ mice, we examined the effects of CRF or ACTH on plasma corticosterone increase (Figure 2). To suppress the release of endogenous ACTH, dexamethasone at 0.1 mg/kg body weight was injected 30 min before ACTH administration. As shown in Figure 2, both CRF and ACTH increased plasma corticosterone levels similarly between PACAP^−/−^ and wild-type mice.

### Attenuated c-Fos expression in the PVN and MeA of PACAP^−/−^ mice after restraint stress

**Figure 3:**
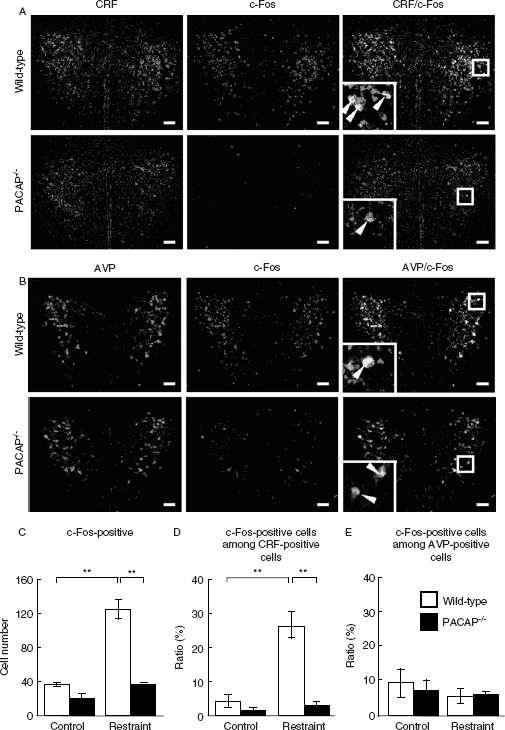
Marked attenuation of restraint stress-induced c-Fos expression in CRF-positive PVN neurons in PACAP^−/−^ mice. PACAP^−/−^ and wild-type mice with and without restraint stress were subjected to double immunofluorescence staining for c-Fos plus CRF (A, C, and D) or AVP (B, C, and E). (A and B) Representative microscope images are shown. Insets, higher magnification images of the regions boxed. Arrowheads indicate double-positive cells. Scale bars, 50 µm. (C–E) The total number of c-Fos-positive cells (C), the fraction of CRH neurons that was c-Fos positive (D), and the fraction of AVP neurons that was c-Fos positive (E) were analyzed in PACAP^−/−^ (closed bars) and wild-type (open bars) mice (*n* = 6–7 per group). **P < 0.01.

Previous studies suggest that the PVN is an integrating center for stress responses (Herman and Cullinan 1997). Therefore, we examined c-Fos expression in the PVN to define the pattern of neurons excited by restraint stress in PACAP^−/−^ mice (Figure 3). After restraint stress for 2h, c-Fos-positive cells were significantly increased by approximately threefold in wild-type mice; however, this increase was absent in PACAP^−/−^ mice (Figure 3A,C). To determine the cell type of c-Fos-positive cells in the PVN, we performed double immunofluorescent staining with the anti-c-Fos antibody together with the anti-CRF or anti-AVP antibody. The numbers of CRF- or AVP-positive neurons were not significantly different between PACAP^−/−^ and wild-type mice as well as between with and without restraint stress (Figure 3A,B; data not shown). After restraint stress, the percentage of c-Fos-positive cells among CRF-positive cells, but not among AVP-positive cells, was significantly increased in wild-type mice; however, such an increase was not observed in PACAP^−/−^ mice (Figure 3A,B,D,E).

**Figure 4:**
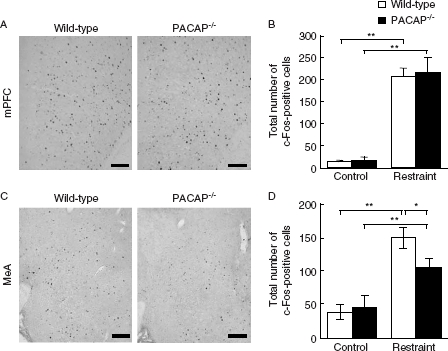
Restraint stress-induced c-Fos expression in the mPFC and MeA in PACAP^−/−^ mice. PACAP^−/−^ and wild-type mice with and without restraint stress were subjected to immunostaining to detect c-Fos expression in the mPFC (A and B) and MeA (C and D). (A and C) Representative microscope images are shown. Scale bars, 100 µm. (B and D) The quantitative results in PACAP^−/−^ (closed bars) and wild-type (open bars) mice are shown (*n* − 6–7 per group). *P < 0.05; **P < 0.01.

It was shown that the activity of the PVN is regulated by other brain regions. For example, the MeA is known to stimulate the PVN, while the mPFC has a negative influence (Herman and Cullinan 1997; Ulrich-Lai and Herman 2009). Therefore, we also examined the MeA and mPFC in PACAP^−/−^ mice after restraint stress. The stress-induced increase in the number of c-Fos-positive cells was significantly attenuated in the MeA in PACAP^−/−^ mice compared with wild-type mice, but the number of c-Fos-positive cells in the mPFC was not different between the two genotypes (Figure 4).

## Discussion

Previous studies demonstrate that PACAP^−/−^ mice show a reduced corticosterone response after trimethyltin (Morita et al. 2006), light exposure after constant dark (Hatanaka et al. 2008), and restraint stress (Stroth and Eiden 2010). Therefore, it may be hypothesized that intrinsic PACAP is crucial for stress response. In the present study, we examined consequences of four different types of acute stressors in PACAP^−/−^ mice, open-field exposure (emotional), cold exposure (metabolic), and ether inhalation (physical), in addition to restraint stress (mainly emotional). We showed that these mice have a markedly attenuated corticosterone increase only after open-field exposure and restraint stress. We also recently showed that plasma glucose increase was greatly attenuated in PACAP^−/−^ mice after the same restraint paradigm (Tanida et al. 2010). Restraint and open-field exposure are categorized as emotional stressors (also described as psychological or processive stressors) that depend on limbic stress pathways involving higher-order sensory processing (Herman and Cullinan 1997). Sensory functions *per se*, however, were not significantly affected in PACAP^−/−^ mice as we examined using SHIRPA, a protocol for comprehensive phenotype assessment (Hatcher et al. 2001). Therefore, the present results suggest that PACAP may be prominently involved in emotional stress-dependent corticosterone responses.

In this study, we examined plasma corticosterone levels only after 4-h cold exposure, because we previously observed that PACAP^−/−^ mice showed greater hypothermia after a 4-h cold exposure compared with wild-type mice, when they were food deprived (data not shown). However, there is a possibility that novel-environment exposure stress precede the metabolic stress in this stress paradigm. If this is the case, it would be expected that an interpretation of the result become difficult. This issue still needs to be carefully examined.

The present observations that PACAP^−/−^ mice normally respond to CRF and ACTH by increasing plasma corticosterone levels as well as previous evidence that PACAP activates CRF gene expression in the rat PVN (Grinevich et al. 1997), indicate that pituitary corticotrope and adrenal cortical functions are normal in terms of corticosterone release. This is not inconsistent with the result that PACAP is involved in upstream activation of the HPA axis and/or has direct effect on pituitary corticotrope (Vaudry et al. 2009).

It was demonstrated that restraint stress induces c-Fos expression in several brain regions, including hypothalamic and amygdaloid nuclei (Chen and Herbert 1995). In the present study, we observed that c-Fos expression in the PVN and MeA was significantly decreased in PACAP^−/−^ mice, whereas that in the mPFC was not affected. It is known that the MeA enhances activation of PVN neurons during immobilization stress (Herman and Cullinan 1997; Ulrich-Lai and Herman 2009). In addition, both PACAP and its receptors are abundantly distributed in the PVN and MeA (Vaudry et al. 2009). Therefore, attenuated restraint stress-induced corticosterone increase in PACAP^−/−^ mice might be attributable to the impaired neuronal response in the PVN and MeA in these mice.

In the present study, we did not examine c-Fos expression in other areas such as extrahypothalamic brain regions (except for mPFC) and other amygdala subregions that regulate the PVN during stressor exposure (Herman and Cullinan 1997; Ulrich-Lai and Herman 2009). This limitation, together with c-Fos expression by stressors that did not produce different responses in PACAP^−/−^ mice clearly needs to be investigated.

c-Fos expression in the control mice that were individually housed before experiments but not exposed to restraint stress was significantly low in both genotypes compared to restrained wild-type mice. Although we do not routinely observe significant c-Fos induction by handling (data not shown), it is still unclear whether the c-Fos measurements reflect the ‘emotional’ aspects of restraint, or whether they just represent handling and placement in the devices. Nevertheless, it is notable that PACAP deficiency led to significantly attenuated c-Fos expression in the PVN and MeA.

CRF and AVP neurons in the PVN are known to convert stress signals into hormonal outputs (Ulrich-Lai and Herman 2009). Here, it was shown that c-Fos and CRF-double positive neurons virtually disappeared in PACAP^−/−^ mice after restraint stress. Hannibal et al. (1995) showed that a subpopulation (44–77%) of PACAP-immunoreactive neurons co-express CRF in the PVN. In the present study, we observed that the rise in corticosterone from 1 h after restraint in PACAP^−/−^ mice was statistically different from wild-type mice. These results may suggest that PACAP directly stimulates CRF neurons in the PVN of which absence is involved in the attenuated corticosterone response in PACAP^−/−^ mice. However, since Hannibal et al. (1995) demonstrated that acute restraint stress for 1 h failed to increase PACAP mRNA expression in the hypothalamus, we cannot exclude the possibility that some component of the CRF/ACTH pathway is PACAP-independent or depends on extrahypothalamic PACAP sources after acute restraint stress. It might be also possible that an inhibitory process is recruited in PACAP^−/−^ mice during restraint and open-field exposure stress that actively inhibits HPA responding.

Several lines of evidence support that PACAP^−/−^ mice are regarded as an animal model of psychiatric conditions (Hashimoto et al. 2001, 2007, 2009, 2010; Tanaka et al. 2006; Hatanaka et al. 2008) and that PACAP signaling might contribute to the pathogenesis of certain psychiatric disorders (Hashimoto et al. 2007, 2010). As it was widely recognized that inadequate and/or prolonged response to stressors may result in psychiatric disorders (Charmandari et al. 2005), impairment of the stress response by altered PACAP signaling systems may partly relate to certain psychiatric conditions and this possibility warrants further investigation in the PVN as corticosterone responses were attenuated in PACAP^−/−^ mice.
